# CHC for pelvic pain in women with endometriosis: ineffectiveness or discontinuation due to side-effects

**DOI:** 10.1093/hropen/hoz040

**Published:** 2020-02-28

**Authors:** Paul J Yong, Najla Alsowayan, Heather Noga, Christina Williams, Catherine Allaire, Sarka Lisonkova, Mohamed A Bedaiwy

**Affiliations:** 1 Department of Obstetrics and Gynaecology, University of British Columbia, Vancouver V6H3N1, Canada; 2 BC Women’s Centre for Pelvic Pain and Endometriosis, Vancouver V6H3N1, Canada; 3 Women’s Health Research Institute, Vancouver V6H3N1, Canada

**Keywords:** combined oral contraceptives, combined hormonal contraception, chronic pelvic pain, endometriosis, pelvic floor myalgia

## Abstract

**STUDY QUESTION:**

What are the use patterns and factors associated with combined hormonal contraception (CHC) ineffectiveness or discontinuation due to side-effects in patients with endometriosis and pelvic pain?

**SUMMARY ANSWER:**

Worse chronic pelvic pain (CPP) severity and pelvic floor myalgia were associated with continuous CHC ineffectiveness, while poorer quality-of-life was associated with continuous CHC discontinuation due to side-effects.

**WHAT IS KNOWN ALREADY:**

CHC is a first line of therapy for endometriosis-associated pelvic pain in women. However, some patients state that CHC is ineffective for their pain, while others have to discontinue CHC due to side-effects.

**STUDY DESIGN, SIZE, DURATION:**

Analysis of a prospective patient database from a tertiary care referral center for patients with endometriosis and pelvic pain between December 2013 and April 2015 was carried out.

**PARTICIPANTS/MATERIALS, SETTING AND METHODS:**

A total of 373 patients of reproductive age with endometriosis from the database were included in the study. Data included patient self-reported questionnaires, physical examination findings and validated instruments. There were four variables of interest: history of cyclical CHC ineffectiveness (yes/no), history of cyclical CHC discontinuation due to side-effects (yes/no), history of continuous CHC ineffectiveness (yes/no) and history of continuous CHC discontinuation due to side-effects (yes/no). The primary outcome was CPP severity for the past 3 months (score of 0–10), and secondary outcomes were other pelvic pain scores, quality-of-life on the Endometriosis Health Profile 30 (EHP-30) and underlying conditions including irritable bowel syndrome, painful bladder syndrome, abdominal wall pain, pelvic floor myalgia and depression, anxiety and pain catastrophizing.

**MAIN RESULTS AND THE ROLE OF CHANCE:**

Among the 373 cases in the dataset, prior cyclical CHC use was reported by 228 (61.1%) women, of which 103 (27.6%) stated it was ineffective for their pain and 94 (25.2%) stated they discontinued CHC due to side-effects. Previous continuous CHC use was reported by 175 (46.9%) women, of which 67 (18.0%) stated it was ineffective and 59 (15.8%) stated they discontinued due to side-effects. Worse CPP severity in the last 3 months was associated with a history of continuous CHC ineffectiveness (*P* < 0.001). Poorer quality-of-life was present in women who reported a history of continuous CHC discontinuation due to side-effects (*P* = 0.005). Among the underlying conditions, pelvic floor tenderness (as a marker of pelvic floor myalgia) was associated with CHC ineffectiveness.

**LIMITATIONS AND REASONS FOR CAUTION:**

This study involved patient recall and no longitudinal follow-up. Also, we do not have data on the type of side-effect that led to discontinuation. Medication ineffectiveness was reported subjectively by the patient rather than using standardized criteria. Finally, the diagnosis of endometriosis was based on previous surgery or a current nodule or endometrioma on examination/ultrasound; without prospective surgical data on all the patients, it was not possible to do a sub-analysis by current surgical features (e.g. stage).

**WIDER IMPLICATIONS OF THE FINDINGS:**

In women with endometriosis, CHC ineffectiveness was associated with worse CPP and pelvic floor myalgia, which suggests myofascial or nervous system contributors to CPP that does not respond to hormonal suppression. A tender pelvic floor, as a sign of pelvic floor myalgia, may be a clinical marker of patients with endometriosis who are less likely to have an optimal response to hormonal suppression. For women who discontinue CHC due to side-effects, research is needed to help alleviate these side-effects as these patients report worse quality-of-life.

**STUDY FUNDING/COMPETING INTEREST(S):**

This work was supported by a Canadian Institutes of Health Research (CIHR) Transitional Open Operating Grant (MOP-142273) as well as BC Women’s Hospital and the Women’s Health Research Institute. PY is also supported by a Health Professional Investigator Award from the Michael Smith Foundation for Health Research. MB/CA has financial affiliations with Abbvie and Allergan; the other authors have no conflicts of interest.

**WHAT DOES THIS MEAN FOR PATIENTS?**

Endometriosis and pelvic pain affect more than 1 in 10 women. In endometriosis, tissue that is similar to the tissue that normally lines the inside of the uterus grows outside the uterus and most commonly involves the ovaries, tubes, and the lining of the pelvis. The birth control pill, which contains female hormones, is commonly used as a treatment for pelvic pain in women with endometriosis. In this study, we asked women with endometriosis and pelvic pain about their experiences of using the birth control pill. For women who said that the birth control pill was not effective for their pain, they reported higher levels of pain and more often had muscle pain. It is therefore important to recognize when women with endometriosis actually have muscle pain, as the birth control pill may not be helpful in their treatment. Women who said that they had to stop the birth control pill because of side-effects reported a worse quality-of-life. Ideally, doctors should find ways to help reduce these side-effects of the birth control pill or find new treatments for these women.

## Introduction

Chronic pelvic pain (CPP) is defined as a constant or intermittent pain in the lower abdominal or pelvic area for a minimum of 6 months, not exclusively associated with menstruation, intercourse, or pregnancy (RCOG, 2012) with a prevalence between 5.7 and 26.6% (Ahangari, 2014). It constitutes a significant challenge for patients and physicians due to its multifactorial complexity and associated psychological morbidities. Endometriosis is a common cause of CPP, with more than 33% of endometriosis patients affected (Guo and Wang, 2006).

Combined hormonal contraception (CHC) is regarded a first line of therapy in treating endometriosis-associated CPP (RCOG, 2012; Dunselman *et al.,* 2014). CHC functions via inhibition of endometriosis lesions and inhibition of gonadal estrogen synthesis through a feedback mechanism on the hypothalamic-pituitary-gonadal axis. The resultant estrogen reduction decreases prostaglandin synthesis, which is a major component in the endometriotic inflammatory response.

Several studies have been performed to evaluate the effectiveness of CHC for endometriosis and/or pelvic pain (Harada *et al.,* 2008; Mabrouk *et al.,* 2011; Ferrari *et al.,* 2012; Tanaka *et al.,* 2016). In a randomized controlled trial comparing the effectiveness of cyclic CHC with placebo for four menstrual cycles, despite significant improvement of dysmenorrhea in the CHC group, there was no significant improvement in the noncyclic pelvic pain scores in the same group. In this trial, four patients (7.8%) discontinued the treatment due to side-effects (Harada *et al.,* 2008).

Some studies have compared the efficacy of cyclic and continuous regimens, and there is evidence for benefit of continuous CHC (Vercellini *et al.,* 2003; Seracchioli *et al.,* 2010a; Vlahos *et al.,* 2013; Caruso *et al.,* 2016). However, randomized (Seracchioli *et al.,* 2010a, 2010b) and nonrandomized (Vercellini *et al.,* 2003; Vlahos *et al.,* 2013) studies have also reported participants’ withdrawal due to the intolerability of CHC side-effects. In addition, some studies reported patients changing the type of CHC used because of bleeding patterns and side-effects (Muzzi *et al.,* 2011; Caruso *et al.,* 2016).

In the current study, we investigated the prevalence of patients with endometriosis reporting a history of CHC ineffectiveness for pain or CHC discontinuation due to side-effects, among women who attended a tertiary referral center for pelvic pain and endometriosis. We also determined whether CHC ineffectiveness or discontinuation due to side-effects was associated with CPP or other pelvic pain measures, as well as quality-of-life, psychological scores and underlying pain conditions.

## Materials and Methods

### Setting

This is an analysis of a prospective database at the BC Women’s Center for Pelvic Pain and Endometriosis, an interdisciplinary tertiary referral center in Vancouver, British Columbia, Canada. This prospective database has been previously described in detail (Yosef *et al.,* 2016; Allaire *et al.,* 2018) and involves prospective consent followed by a series of online patient questionnaires completed prior to the initial visit, validated psychological and quality-of-life instruments, and real-time entry of data from the gynecologist assessment.

### Ethics approval

Ethics approval was granted by the University of British Columbia (H11-02882).

### Study population

In this study, we included participants who were referred or re-referred to our center between December 2013 and April 2015, and who had a diagnosis of endometriosis (previous surgical diagnosis, current nodule on examination, or current endometrioma on ultrasound). Exclusion criteria were participants older than 50 years or postmenopausal status.

### Variables of interest

Participants were asked to retrospectively recall whether they have ever taken CHC for their pain (including both past and current use), and whether CHC was taken cyclically and/or continuously. For those who reported having taken CHC cyclically, participants were asked whether CHC was ineffective for their pain and/or whether the CHC was discontinued due to side-effects. Similarly, for those who reported having taken CHC continuously, participants were asked whether CHC was ineffective for their pain and/or whether the CHC was discontinued due to side-effects.

Thus, there were four variables of interest: history of cyclical CHC ineffectiveness (yes/no), history of cyclical CHC discontinuation due to side-effects (yes/no), history of continuous CHC ineffectiveness (yes/no) and history of continuous CHC discontinuation due to side-effects (yes/no). Based on the nature of the questions, it was possible for patients to have tried both cyclical and continuous CHC, and to answer ‘yes’ to both ineffectiveness and discontinuation due to side-effects.

### Analyses for primary and secondary outcomes

Primary outcome was CPP severity for the past 3 months assessed using an 11-point numeric rating scale (0–10). Secondary outcomes were other pain symptoms in the past 3 months (dysmenorrhea, dyschezia and back pain) (11-point scale), ever symptoms of superficial and deep dyspareunia (11-point scale), and quality of life in the last 4 weeks via the pain subscale of the Endometriosis Health Profile 30 (EHP-30) (Jones *et al.,* 2001).

Bivariate association was performed between each of the four variables of interest, and the primary and secondary outcomes. For the primary outcome, logistic regression modeling was also carried out, controlling for demographic factors, followed by sequential backward elimination until all variables in the regression model had a *P* value below the removal threshold (*P* < 0.05).

### Corollary analyses

We also sought to determine whether the four variables of interest were associated with underlying conditions, which could explain the association with the primary/secondary outcomes. The underlying conditions were a previous surgical confirmation of endometriosis; diagnosis of irritable bowel syndrome (IBS) using the Rome III criteria (Drossman, 2006); diagnosis of painful bladder syndrome (PBS) using the criteria of the American Urological Association or International Continence Society (Meijlink, 2014); abdominal wall pain, typically due to myofascial trigger points, and diagnosed by positive Carnett test (Yosef *et al.,* 2016); bladder or pelvic floor tenderness on pelvic examination as a marker of pelvic floor myalgia (Yosef *et al.,* 2016); and moderate depression assessed by a Patient Health Questionnaire (PHQ-9) score ≥ 10 (Kroenke *et al.,* 2001), moderate anxiety assessed by a generalized anxiety disorder (GAD-7) questionnaire score ≥ 10 (Spitzer *et al.,* 2006), and catastrophizing assessed by the pain catastrophizing scale score ≥ 30 (75th centile) (Sullivan *et al.,* 1995).

In addition, we determined whether a history of CHC ineffectiveness or discontinuation was associated with features of endometriosis: Stage I–II versus Stage III/IV in those who had previous surgery, presence versus absence of a nodule at the initial visit at the center and presence versus absence of an endometrioma at the initial visit at the center.

### Statistical analysis

Analysis was performed using SPSS software (SPSS, V22.0; IBM Corp, Armonk, NY, USA) with *P* value <0.05 considered to show significance. Means were provided with SD, medians with ranges and odds ratio with CI. Bivariate testing was carried out using the two sample Student’s *t*-test (with Welch’s correction if a significant difference in variances using Levene’s test), Fisher’s Exact test, or Chi-square test, depending on the variables involved. The proportion of missing values in anxiety, depression and pain catastrophizing variables were 5.6% in the total cohort and thus were imputed as previously reported (Yosef *et al.,* 2016).

**Table I:** **Demographic characteristics of participants in the four groups of interest**.

Variable	Cyclic CHC ineffective			Cyclic CHC discontinued due to side effects			Continuous CHC ineffective			Continuous CHC discontinued due to side effects		
	Yes (*n* = 103)	No (*n* = 125)	*P*	Yes (*n* = 94)	No (*n* = 134)	*P*	Yes (*n* = 67)	No (*n* = 108)	*P*	Yes (*n* = 59)	No (*n* = 116)	*P*
Age (years) mean ± SD	31.7 ± 6.5	34.5 ± 7.4	0.00^*^	34.1 ± 7.0	32.6 ± 7.1	0.12	30.0 ± 5.8	33.8 ± 7.4	0.00^*^	33.3 ± 7.2	31.9 ± 6.9	0.21
BMI (kg/m^2^) mean ± SD	25.4 ± 5.5	25.2 ± 5.8	0.75	25.5 ± 6.2	25.1 ± 5.2	0.56	25.5 ± 5.7	25.1 ± 5.7	0.63	25.2 ± 4.6	25.3 ± 6.2	0.98
Married	31 (31.0%)	56 (45.5%)	0.03^*^	37 (39.8%)	50 (38.5%)	0.89	19 (29.7%)	44 (41.9%)	0.14	23 (39.0%)	40 (36.4%)	0.74
Smoker	11 (11.0%)	14 (11.4%)	1.0	11 (11.8%)	14 (10.8%)	0.83	6 (9.4%)	15 (14.3%)	0.47	8 (13.6%)	13 (11.8%)	0.81
Parous	25 (25.0%)	35 (28.5%)	0.65	26 (28.0%)	34 (26.2%)	0.76	12 (18.8%)	27 (25.7%)	0.35	18 (30.5%)	21 (19.1%)	0.13
**Education**												
Some high school	2 (2.0%)	5 (4.1%)		3 (3.2%)	4 (3.1%)		2 (3.1%)	3 (2.9%)		1 (1.7%)	4 (3.6%)	
High school	11 (11.0%)	11 (8.9%)		12 (12.9%)	10 (7.7%)		5 (7.8%)	15 (14.3%)		10 (16.9%)	10 (9.1%)	
Some college	26 (26%)	29 (23.6%)		26 (28.0%)	29 (22.3%)		15 (23.4%)	20 (19.0%)		11 (18.6%)	24 (21.8%)	
Graduated 2 year college	19 (19%)	20 (16.3%)	0.82	11 (11.8%)	28 (21.5%)	0.27	12 (18.8%)	17 (16.2%)	0.79	8 (13.6%)	21 (19.1%)	0.46
Graduated 4 year college	22 (22.0%)	24 (19.5%)		21 (22.6%)	25 (19.2%)		15 (23.4%)	22 (21.0%)		15 (25.4%)	22 (20.0%)	
Post graduate	17 (17%)	30 (24.4%)		19 (20.4%)	28 (21.5%)		11 (17.2%)	24 (22.9%)		13 (22.0%)	22 (20.0%)	
Other	3 (3.0%)	4 (3.3%)		1 (1.1%)	6 (4.6%)		4 (6.3%)	4 (3.8%)		1 (1.7%)	7 (6.4%)	
**Annual income (Canadian Dollars)**												
Less than 20 000	13 (13.0%)	11 (8.9%)	0.09	11 (11.8%)	13 (10.0%)	0.46	12 (18.8%)	7 (6.7%)	0.04^*^	8 (13.6%)	11 (10.0%)	0.26
20 000–39 999	18 (18.0%)	21 (17.1%)		18 (19.4%)	21 (16.2%)		15 (23.4%)	16 (15.2%)		7 (11.9%)	24 (21.8%)	
40 000–59 999	17 (17.0%)	13 (10.6%)		8 (8.6%)	22 (16.9%)		8 (12.5%)	13 (12.4%)		7 (11.9%)	14 (12.7%)	
60 000–79 999	25 (25.0%)	22 (17.9%)		20 (21.5%)	27 (20.8%)		13 (20.3%)	19 (18.1%)		13 (22.0%)	19 (17.3%)	
80 000–99 999	11 (11.0%)	18 (14.6%)		15 (16.1%)	14 (10.8%)		5 (7.8%)	16 (15.2%)		11 (18.6%)	10 (9.1%)	
100 000 or more	16 (16.0%)	38 (30.9%)		21 (22.6%)	33 (25.4%)		11 (17.2%)	34 (32.4%)		13 (22.0%)	32 (29.1%)	

^*^Significant *P* value; 0.00 ≤ 0.009; statistical tests included the two sample Student’s *t*-test (with Welch’s correction if a significant difference in variances using Levene’s test), Fisher’s Exact test or Chi-square test, depending on the variables involved. Proportions do not add up to 100% due to missing values. CHC: combined hormonal contraception.

## Results

### Descriptive statistics

**Figure 1:**
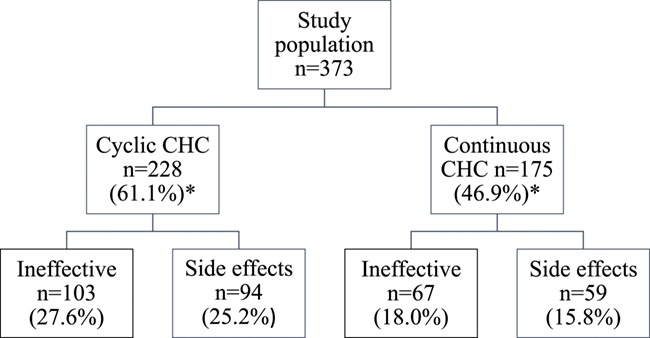
**Proportion of cases with ineffectiveness or discontinuation due to side-effects for cyclic versus continuous use of CHC in patients with endometriosis and pelvic pain.** Note that a given patient could be in more than one category. CHC: combined hormonal contraception.

A total of 373 patients from the database met the study criteria (Fig. 1). Of the total, 343 had a previous surgical diagnosis of endometriosis; where staging was available (*n* = 303), an equal proportion were Stage I–II (46.5%; 141/303) and Stage III/IV (53.5%; 162/303). The remaining 30 did not have previous surgery, but had a diagnosis of endometriosis through the identification of a nodule or endometrioma on examination/ultrasound at the initial visit at the center. A total of 73 participants had a nodule and 88 participants had an endometrioma at the initial visit (i.e. some individuals with previous surgery had recurrence of disease).

In the study population, 228 (61.1%) reported prior use of cyclic CHC, while 175 participants (46.9%) reported history of using continuous CHC (some patients were in both categories).

For the four variables of interest: 103 (27.6% of the study sample) reported cyclical CHC ineffectiveness, 94 (25.2%) reported cyclical CHC discontinuation due to side-effects, 67 (18.0%) reported continuous CHC ineffectiveness and 59 (15.8%) reported continuous CHC discontinuation due to side-effects (Fig. 1).

Demographic characteristics and comparisons are summarized in Table I. Those who reported cyclic CHC to be ineffective were younger (*P* = 0.004) and less likely to be married (*P* = 0.028). Those who reported continuous CHC to be ineffective were also significantly younger (*P* < 0.001) and had lower incomes (*P* = 0.036). No differences were noted for those who reported discontinuing the cyclic or combined CHC due to side-effects (Table I).

### Analyses for primary and secondary outcomes

Associations between the four variables of interest, and the primary/secondary outcomes, are summarized in Table II.

**Table II:** **Ineffectiveness or discontinuation of CHC**.

Cyclic CHC	Ineffective			Discontinued due to side effects		
	Yes (*n* = 103)	No (*n* = 125)	*P*	Yes (*n* = 94)	No (*n* = 134)	*P*
CPP (0–10)	6.3 ± 3.1	5.8 ± 3.0	0.23	6.3 ± 2.7	5.7 ± 3.3	0.15
Dysmenorrhea^a^ (0–10)	7.7 ± 2.1	6.9 ± 2.5	0.03^*^	7.6 ± 2.1	6.9 ± 2.5	0.04
Superficial dyspareunia^b^ (0–10)	4.6 ± 3.1	3.8 ± 3.3	0.05	3.8 ± 3.4	4.4 ± 3.1	0.17
Deep dyspareunia^b^ (0–10)	6.5 ± 3.0	5.7 ± 3.1	0.06	6.1 ± 3.2	6.0 ± 3.0	0.75
Dyschezia (0–10)	4.8 ± 3.1	4.6 ± 3.2	0.64	4.9 ± 3.1	4.6 ± 3.1	0.43
Back pain (0–10)	5.6 ± 3.3	5.5 ± 2.8	0.83	5.7 ± 2.9	5.4 ± 3.1	0.41
Quality of life (EHP-30) (0–100)	50.4 ± 23.4	48.3 ± 23.8	0.53	51.8 ± 22.6	47.3 ± 24.2	0.17
**Continuous CHC**	**Ineffective**			**Discontinued due to side effects**		
	**Yes (*n* = 67)**	**No (*n* = 108)**	***P***	**Yes (*n* = 59)**	**No (*n* = 116)**	***P***
CPP (0–10)	7.1 ± 2.6	5.2 ± 3.3	0.00^*^	6.4 ± 2.7	5.7 ± 3.4	0.13
Dysmenorrhea^a^ (0–10)	7.8 ± 2.1	6.8 ± 2.7	0.03^*^	7.6 ± 2.2	6.8 ± 2.7	0.09
Superficial dysareunia^b^ (0–10)	4.8 ± 3.5	3.8 ± 3.2	0.06	3.7 ± 3.3	4.5 ± 3.3	0.15
Deep dyspareunia^b^ (0–10)	7.0 ± 2.8	6.0 ± 3.0	0.03^*^	6.5 ± 2.9	6.3 ± 3.0	0.71
Dyschezia (0–10)	5.4 ± 3.0	4.3 ± 3.3	0.03^*^	4.7 ± 3.1	4.7 ± 3.3	0.98
Back pain (0–10)	5.9 ± 3.3	5.2 ± 2.8	0.15	5.9 ± 2.7	5.3 ± 3.2	0.19
Quality of life (0–100)	52.1 ± 23.5	47.2 ± 24.9	0.21	55.8 ± 19.4	45.6 ± 26.1	0.00^*^

a ^a^Excludes those amenorrheic.

b ^b^Excludes those never sexually active.

^*^Significant *P* value; 0.00 ≤ 0.009; statistical test was the two sample Student’s *t*-test (with Welch’s correction if a significant difference in variances using Levene’s test).

CPP: chronic pelvic pain.

For the primary outcome of CPP, those who reported continuous CHC being ineffective had a higher severity of CPP in the last 3 months (*P* < 0.001) (Table II). Multiple logistic regression was performed, which showed that the association between CPP and CHC ineffectiveness was independent of demographic variables (age, marital status, and income) (Table III).

**Table III:** **Binary logistic regression for CHC ineffectiveness**.

Variable	Odds ratio^a^	95% CI	*P* value
**Continuous CHC ineffective**			
CPP (0–10)	1.20	1.06–1.35	0.003^*^
Age	0.95	0.90–1.00	0.049^*^
Marital status	1.54	0.65–3.62	0.33
Annual income	0.75	0.60–0.95	0.015^*^

a Odds ratio is derived from exponential (B) value.

^*^Significant *P* value, Wald test.

For secondary outcomes (Table II), continuous CHC ineffectiveness was also associated with higher severity of dysmenorrhea (*P* = 0.025), deep dyspareunia (*P* = 0.030), and dyschezia (*P* = 0.025). Cyclic CHC ineffectiveness and discontinuation due to side-effects were associated with worse dysmenorrhea (*P* = 0.029, *P* = 0.043). Those who reported continuous CHC discontinuation due to side-effects had poorer quality-of-life (*P* = 0.005).

### Corollary analyses

In Table IV, we show the associations between the four variables of interest and underlying conditions. Pelvic floor tenderness was significantly associated with cyclic and continuous CHC ineffectiveness (*P* = 0.003 and *P* < 0.001, respectively), and PBS was also associated with continuous CHC ineffectiveness (*P* = 0.043). There were no associations with IBS, abdominal wall pain or psychological measures.

**Table IV:** **Association between CHC ineffectiveness or side effects and underlying conditions**.

Diagnosis	Cyclic CHC ineffective			Cyclic CHC discontinued due to side effects			Continuous CHC ineffective			Continuous CHC discontinued due to side effects		
	Yes (*n* = 103)	No (*n* = 125)	*P*	Yes (*n* = 94)	No (*n* = 134)	*P*	Yes (*n* = 67)	No (*n* = 108)	*P*	Yes (*n* = 59)	No (*n* = 116)	*P*
IBS	56 (54.4%)	73 (58.4%)	0.59	57 (60.6%)	72 (53.7%)	0.34	35 (52.2%)	63 (58.3%)	0.44	32 (54.2%)	66 (56.9%)	0.75
Painful bladder syndrome	47 (45.6%)	56 (44.8%)	1.0	43 (45.7%)	60 (44.8%)	0.89	39 (58.2%)	45 (41.7%)	0.04	29 (49.2%)	55 (47.4%)	0.87
Abdominal wall pain (Carnett test)	49 (47.6%)	43 (34.4%)	0.06	39 (41.5%)	53 (39.6%)	0.79	31 (46.3%)	37 (34.3%)	0.15	25 (42.4%)	43 (37.1%)	0.52
Bladder tenderness	29 (28.2%)	22 (17.9%)	0.08	24 (26.1%)	27 (20.1%)	0.33	20 (29.9%)	19 (17.9%)	0.09	18 (31.6%)	21 (18.1%)	0.05
Pelvic floor tenderness	44 (42.7%)	29 (23.6%)	0.00^*^	31 (33.7%)	42 (31.3%)	0.77	32 (47.8%)	21 (19.8%)	0.00^*^	20 (35.1%)	33 (28.4%)	0.39
Depression (Patient Health Questionnaire-9 ≥ 10)	37 (35.9%)	42 (33.6%)	0.78	34 (36.2%)	45 (33.6%)	0.78	28 (41.8%)	38 (35.2%)	0.43	27 (45.8%)	39 (33.6%)	0.14
Anxiety (Generalized Anxiety Disorder questionnaire-7 ≥ 10)	27 (26.2%)	33 (26.4%)	1.0	25 (26.6%)	35 (26.1%)	1.0	20 (29.9%)	29 (26.9%)	0.73	20 (33.9%)	29 (25.0%)	0.22
Pain catastrophizing scale ≥30 (75th centile)	31 (30.1%)	29 (23.2%)	0.29	28 (29.8%)	32 (23.9%)	0.36	20 (29.9%)	23 (21.3%)	0.21	18 (30.5%)	25 (21.6%)	0.20

^*^Significant *P* value; 0.00 < 0.009; Fisher Exact test. Numbers and proportions (%) represent the patients with IBS among those with cyclic CHC ineffectiveness and among those without cyclic CHC ineffectiveness, etc.

IBS: irritable bowel syndrome.

In Table V, associations with endometriosis features are shown. In general, CHC ineffectiveness or discontinuation was associated with less severe disease, with the following reaching statistical significance: cyclic CHC ineffectiveness and continuous CHC discontinuation associated with the absence of endometrioma (*P* = 0.041, *P* = 0.021) and Stage I–II disease (*P* = 0.008, *P* = 0.022).

**Table V:** **Association between CHC ineffectiveness or side effects and endometriosis features**.

Endometriosis features	Cyclic CHC ineffective			Cyclic CHC discontinued due to side effects			Continuous CHC ineffective			Continuous CHC discontinued due to side effects		
	Yes	No	*P*	Yes	No	*P*	Yes	No	*P*	Yes	No	*P*
Stage III/IV disease at previous surgery	26/86 (30.2%)	51/103 (49.5%)	0.01	27/77 (35.1%)	50/112 (44.6%)	0.23	21/53 (39.6%)	44/93 (47.3%)	0.39	15/49 (30.6%)	50/97 (51.5%)	0.02
Current nodule at initial visit	14/103 (13.6%)	21/123 (17.1%)	0.58	13/92 (14.1%)	22/134 (16.4%)	0.71	10/67 (14.9%)	21/106 (19.8%)	0.54	8/57 (14.0%)	23/116 (19.8%)	0.41
Current endometrioma at initial visit	10/100 (10.0%)	25/122 (20.5%)	0.04	12/88 (13.6%)	23/134 (17.2%)	0.57	9/65 (13.8%)	17/104 (16.3%)	0.83	3/54 (5.6%)	23/115 (20.0%)	0.02

^*^Significant *P* value; 0.00 < 0.009; Fisher Exact test. Numbers and proportions (%) represent the patients with Stage III/IV disease among those with cyclic CHC ineffectiveness and among those without cyclic CHC ineffectiveness, etc.

## Discussion

In this study of women with endometriosis, 18–28% stated that CHC was ineffective for their pain, and 15–25% discontinued CHC due to side-effects. Our results are consistent with the findings of Becker *et al.* (2017) in a systematic review of endometriosis medical therapies, where they reported a 5–24% rate of discontinuation of CHC. In addition, we found that worse CPP severity was associated with a history of continuous CHC ineffectiveness, while poorer quality-of-life was associated with a history continuous CHC discontinuation due to side-effects.

The main strengths of the study are the use of prospectively consented patients in a large database, and the inclusion of physical examination findings, standardized diagnostic criteria and validated instruments. One limitation is that although patients were prospectively consented for inclusion in the database, this study involved data from the patients’ retrospective recall. In addition, no data were collected on the type of side-effects experienced by the patient, and the medication ineffectiveness was reported subjectively by the patient rather than using standardized criteria. Finally, the diagnosis of endometriosis was based on previous surgery or a current nodule or endometrioma on examination/ultrasound; without prospective surgical data on all the patients, it was not possible to do a sub-analysis by current surgical features (e.g. stage).

The association between continuous CHC ineffectiveness and worse CPP may be due to the multifactorial pathophysiology of CPP, which includes nervous system and myofascial mechanisms (Stratton *et al.,* 2015) that would not be directly treated with CHC. Notably, we found that pelvic floor tenderness (as a marker of pelvic floor myalgia) was associated with CHC ineffectiveness. This points to the importance of pelvic floor dysfunction in CPP (Yosef *et al.,* 2016), and may explain why CHC was ineffective in patients reporting more severe CPP. A weaker association between PBS and continuous CHC ineffectiveness was also observed, which is consistent with the known association between PBS and pelvic floor dysfunction (Yong *et al.,* 2017). Therefore, pelvic floor myalgia (tender pelvic floor) may be a clinical marker for those patients with endometriosis who are less likely to have an optimal response to hormonal suppression and in whom nongynecologic and multidisciplinary treatments should be sought. However, it should be emphasized that the cross-sectional nature of the study does not allow us to establish cause and effect. Therefore, the association between CHC ineffectiveness and pelvic floor myalgia remains a correlation only, and requires prospective data to determine whether pelvic floor myalgia can predict lack of response to CHC.

The association between continuous CHC discontinuation due to side-effects and poorer quality-of-life suggests that the CHC treatment regimen could have been effective for these patients, but they had to stop the CHC and thus lost the potential benefit, which ultimately resulted in a worse quality of life. Alternatively, patients with more chronic pain and nervous system sensitization (who also have worse quality-of-life on average) tend to have side-effects with a variety of medications and environmental exposures, and thus they are also less likely to tolerate the side-effects of CHC.

Another interesting observation was the association between continuous CHC ineffectiveness and lower annual household income. Our study was conducted in a population with a universal province-wide health care plan, but not a universal pharmacare plan, and we do not know whether an individual patient in the study paid for CHC out of pocket or had coverage through third-party insurance. Several studies have identified that female gender, lower income, chronic conditions, and increased drug cost or decreased insurance co-payment can significantly affect patient adherence to treatment (Jin *et al.,* 2008; Eaddy *et al.,* 2012; Cutler *et al.,* 2018). In addition, a Canadian study identified living in British Columbia as a risk factor for lack of adherence to medications compared to other provinces (Law *et al.,* 2012). Another cross-sectional study assessing the cost related to treatment nonadherence in 11 developed countries concluded that Canada has the second highest national prevalence of cost-related nonadherence (the first being the USA), although this study focused on patients older than 55 years old (Morgan and Lee, 2017).

Continuous CHC ineffectiveness was also associated with secondary pain outcomes, such as worse dysmenorrhea, deep dyspareunia and dyschezia. This suggests that pelvic floor myalgia (with or without PBS) may also be associated with these pain symptoms, in addition to CPP. Cyclic CHC ineffectiveness and discontinuation were associated with worse dysmenorrhea, but not with CPP. This raises the question of why continuous CHC ineffectiveness, but not cyclic CHC ineffectiveness, was associated with worse CPP. One explanation may be lack of power, as there was still a nonsignificant trend toward worse CPP in those with cyclic CHC ineffectiveness. An alternative explanation may be that cyclic CHC ineffectiveness is due in part to decreased suppression (during the pill-free week), which would be associated with dysmenorrhea, but not necessarily the myofascial and central nervous system mechanisms underlying CPP.

We also found that CHC ineffectiveness and discontinuation due to side-effects were associated with less severe endometriosis disease, in particular the absence of ovarian endometrioma at the initial visit at the center and Stage I–II disease at the time of previous surgery. A possible explanation is that those with more severe endometriosis may be expected to have primarily gynecologic pain that is responsive to hormonal suppression, while those with less severe endometriosis may be more likely to have nervous system or musculoskeletal factors involved in their pain pathophysiology and thus are more likely to report CHC ineffectiveness.

In our current research, we are now longitudinally following these patients to assess CHC use patterns prospectively. Future research into the alleviation of CHC side-effects in women with pelvic pain is also important, as these patients report worse quality-of-life; for example, CHC-related mood side-effects could be managed with psychological or pharmacological therapy. Another future research endeavor would be a clinical trial of CHC for pelvic pain, where patients are stratified by the presence of gynecologic and nongynecologic pain generators, in order to confirm that CHC is less effective in those with pelvic floor myalgia.
